# Activity of the upstream TATA-less promoter of the *p21*^*Waf1/Cip1*^ gene depends on transcription factor IIA (TFIIA) in addition to TFIIA-reactive TBP-like protein

**DOI:** 10.1111/febs.12848

**Published:** 2014-06-06

**Authors:** Hidefumi Suzuki, Ryo Maeda, Tomoyoshi Nakadai, Taka-aki Tamura

**Affiliations:** Graduate School of Science, Chiba UniversityJapan

**Keywords:** p21, p53, TFIIA, TLP, transcription

## Abstract

TATA-binding protein-like protein (TLP) binds to transcription factor IIA (TFIIA) with high affinity, although the significance of this binding is poorly understood. In this study, we investigated the role of TFIIA in transcriptional regulation of the *p21*^*Waf1/Cip1*^ (*p21*) gene. It has been shown that TLP is indispensable for p53-activated transcription from an upstream TATA-less promoter of the *p21* gene. We found that mutant TLPs having decreased TFIIA-binding ability exhibited weakened transcriptional activation function for the upstream promoter. Activity of the upstream promoter was enhanced considerably by an increased amount of TFIIA in a p53-dependent manner, whereas activity of the TATA-containing downstream promoter was enhanced only slightly. TFIIA potentiated the upstream promoter additively with TLP. Although TFIIA is recruited to both promoters, activity of the upstream promoter was much more dependent on TFIIA. Recruitment of TFIIA and TLP to the upstream promoter was augmented in etoposide-treated cells, in which the amount of TFIIA–TLP complex is increased, and TFIIA-reactive TLP was required for the recruitment of both factors. It was confirmed that etoposide-stimulated transcription depends on TLP. We also found that TFIIA-reactive TLP acts to decrease cell growth rate, which can be explained by interaction of the *p21* promoter with the transcription factors that we examined. The results of the present study suggest that the upstream TATA-less promoter of *p21* needs TFIIA and TFIIA-reactive TLP for p53-dependent transcriptional enhancement.

**Structured digital abstract:**

- TLP physically interacts with TFIIA beta and TFIIA alpha by anti tag coimmunoprecipitation (View interaction)
- TFIIA alpha/beta physically interacts with TLP by anti bait coip (View interaction)

## Introduction

Transcriptional regulation of an RNA polymerase II driven gene is governed by a particular set of gene-specific DNA-reactive transcription regulatory factors and their associating transcriptional cofactors. Moreover, general transcription factors such as TFIID (transcription factor IID) and TFIIA are assembled at a promoter region to conduct transcriptional initiation [1–3], which is enhanced by gene-specific transcription regulatory factors through functional interaction.

TATA-binding protein (TBP) is an essential component in TFIID that binds to the TATA-box promoter element [2,4–6]. TBP-like protein (TLP, also called TRF2) has been identified as one of the TBP family proteins [7–9] and has been shown to enhance expression of TATA-less genes such as *NF-1*, *Cyclin-G2*, *TAp63* and *Wee1* [10–12]. TLP is unable to bind to the TATA-box, and a consensus TLP-binding sequence has not been determined so far. In the *Drosophila PCNA* gene, TLP is engaged in transcriptional activation as a cofactor for a transcription regulatory factor called DREF [13]. Although TLP is usually concentrated in the cytoplasm, it translocates to the nucleus in a particular cell-cycle period or when activated by a genotoxin such as etoposide [12]. Hence, TLP is thought to be involved in gene regulation related to growth control and DNA damage response. Recently, we have identified *p21* (*p21*^*Waf1/Cip1*^) as one of the TLP-target genes [14].

p21 is a CDK inhibitor and causes cell-cycle arrest at G_1_ or G_2_ phase [15,16]. Since p21 also participates in apoptosis, DNA repair and tumor suppression in some cases [17], *p21* is regarded as a major gene for cell growth regulation. The amount of intracellular p21 is regulated at the transcription level, and its expression level is enhanced by multiple transcription factors [18,19]. The promoter-enhancer region of *p21* contains several binding sites for p53, which enhances the promoter activity. p53, which works for genome homeostasis, is a typical tumor suppressor and major regulator of the *p21* gene [20–22]. The human *p21* gene has two major promoters: a TATA-containing downstream promoter and an upstream TATA-less promoter [23,24]. TFIID is recruited to the TATA-box of the downstream promoter together with p53 upon UV irradiation, but it does not participate in regulation of the upstream promoter [24]. We have found that activity of the upstream promoter absolutely depends on TLP and p53 [14], and these two factors form a complex in cells [14,25].

The most attractive property of TLP is its potent TFIIA-binding ability. TFIIA is another member of the family of general transcription factors [3]. Although TFIIA binds to TBP to some extent in order to potentiate TFIID-dependent promoters, it is also used for TATA-less promoters [3,26,27]. In higher eukaryotes, TFIIA consists of three subunits including TFIIA α, β and γ [3]. TFIIAαβ is encoded by a single gene and is cleaved into individual α and β subunits [3,28]. We have found that TLP binds more strongly to TFIIA than to TBP [29]. Although TLP is mainly localized in the cytoplasm, mutant TLPs with impaired TFIIA-binding ability display a diffuse localization pattern [29]. However, the significance of the TFIIA-binding ability of TLP in transcriptional regulation has remained to be clarified.

In this study, we investigated the contribution of TLP–TFIIA interaction to *p21* gene regulation, and we found that mutant TLPs with weakened TFIIA-binding ability exhibit decreased transcription stimulation activity. Moreover, etoposide, which stimulates *p21* gene expression, facilitated binding of the upstream promoter to TFIIA and TFIIA-reactive TLP. One reason why TLP possesses a strong TFIIA-binding ability may be elucidated through this study.

## Results

### Transcriptional activation function of mutant TLPs for the p21 promoter

**Fig. 1:**
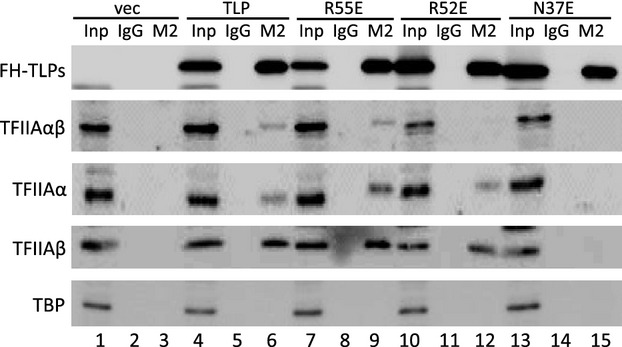
TFIIA-binding ability of TLP. Co-immunoprecipitation to detect the interaction between TLP and TFIIA. Extracts of HCT116 cells into which FH-TLP (TLP) and its mutants (R55E, R52E and N37E) had been introduced were immunoprecipitated with M2 beads (M2) and examined for indicated proteins by western blotting using specific antibodies. Inp, input.

We previously constructed various kinds of mutant TLPs [29]. Among them, N37E and R52E have weakened binding ability to TFIIA, and N37E is a more severe mutant than R52E for TFIIA binding, whereas R55E binds to TFIIA as strongly as does wild-type TLP. In this study, we first investigated in detail the intracellular TFIIA-binding strength of these mutants by co-immunoprecipitation assays. It was confirmed that wild-type TLP and R55E exhibited significant binding to TFIIA, whereas N37E did not bind to TFIIA (Fig. 1). We further investigated processed and unprocessed forms of TFIIAα/β and found that R52E binds to the α and β subunits of TFIIA but does not bind to uncleaved TFIIAαβ (Fig. 1, lane 12).

**Fig. 2:**
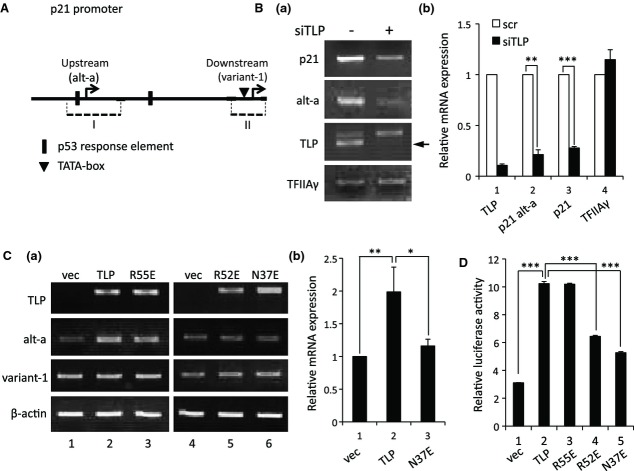
Activation of the endogenous *p21* upstream promoter by TFIIA-reactive TLP. (A) Schematic representation of the promoter region of the human *p21* gene. Transcription start sites for the upstream and downstream promoters, which produce alt-a and variant-1 transcripts, respectively, are shown by arrows. Dotted lines (I and II) represent promoter regions included in luciferase reporter plasmids. (B) Effect of TLP knockdown on *p21* gene expression. Amounts of whole *p21* transcripts (p21) and alt-a (alt-a, p21 alt-a) in HeLa cells were determined by semi-quantitative RT-PCR (a) and RT-qPCR (b). Control siRNA is depicted as – or scr. Each mRNA level for scr (open column) is assigned as 1.0, and relative mRNA level for siTLP (solid columns) is shown. Arrow, position of the specific signal. (C) Effects of overexpressed TLP and its mutants on mRNA transcribed from upstream (alt-a) and downstream (variant-1) promoters. HCT116 cells into which TLP or its mutants had been introduced were assayed for *p21* transcripts by RT-PCR (a) and RT-qPCR (b) using specific primer sets. The relative mRNA levels for TLP and N37E are displayed as ratios to the mRNA level for vec (lane 1). vec, empty vector. (D) Transcriptional activation of the upstream promoter by mutant TLPs. HCT116 cells transfected with indicated effector plasmids together with a reporter plasmid (p21up/GL4) carrying *p21* promoter-containing DNA from −2266 to −1875 were examined by luciferase assay for transcriptional activation function of the native and mutant TLPs.

The human *p21* gene produces mainly alt-a and variant-1 transcripts from the upstream and downstream promoters, respectively (Fig. 2A) [14,23]. Knockdown of endogenous TLP resulted in decreased production of whole *p21* transcripts, mainly due to the decreased level of alt-a transcripts (Fig. 2B). An overexpression experiment confirmed that alt-a is specifically dependent on intracellular TLP level. Next, we investigated the transcriptional activation function of the above-mentioned TLP mutants. R55E, which has a native TFIIA-binding ability, considerably enhanced alt-a production (Fig. 2C–a, lane 3), whereas R52E and N37E exhibited almost no effect (lanes 5 and 6). R52E and N37E showed decreased transcription stimulation activity for the upstream promoter compared with that of wild-type TLP and R55E (Fig. 2C). To obtain direct evidence that TFIIA-reactive TLP potentiates the upstream promoter, we performed a luciferase reporter assay in mutant TLP-overexpressed cells. It was demonstrated that R55E enhanced promoter activity as much as wild-type TLP did, whereas R52E and N37E had less effect on promoter activity than did wild-type TLP (Fig. 2D). These results suggest that TFIIA-binding ability is required for the transcription stimulation function of TLP.

### TFIIA sensitivity of the upstream promoter

**Fig. 3:**
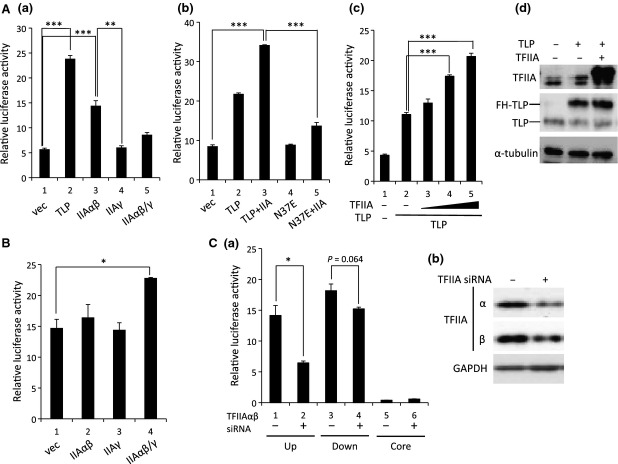
Activation of *p21* promoter by TFIIA. The promoter activity in a reporter plasmid was examined in response to TFIIA in normal HCT116 cells. Cells were co-transfected with TFIIA expression plasmids for TFIIAαβ (IIAαβ, IIA) and TFIIAγ (IIAγ), and activities of the upstream promoter in p21up/GL4 (A) and downstream promoter in p21down/GL4 (B) were determined. (A-b) Cells were transfected with TLP and TFIIAαβ or N37E and TFIIAαβ to investigate the additive effect of the transcription factors. (A-c) Cells were transfected with a constant amount of TLP and an increasing amount of TFIIAαβ. (A-d) Expression levels of TFIIA and TLP protein were determined by western blotting. (C) Cells were co-transfected with TFIIAαβ siRNA (+) or control siRNA (−) and the indicated reporter plasmids. (C-a) Luciferase activities were determined for the upstream (up) and downstream (down) promoters; core, core region of the downstream promoter without a TATA-box in p21core/GL4 plasmid. (C-b) Effect of TFIIA knockdown was checked by western blotting.

**Fig. 4:**
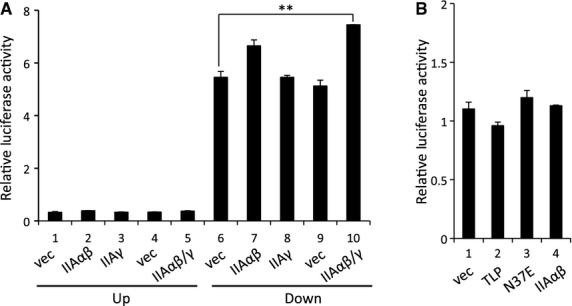
p53-dependent promoter activation by TFIIA. Promoter activation function of TFIIA was examined in a p53-deficient condition. (A) Experiments similar to those for which results were shown in Fig. 3A-b, B were performed using *p53*^−/−^ HCT116 cells, and activities of upstream and downstream promoters were determined. (B) Cells were transfected with expression plasmids of TLP, N37E and TFIIAαβ and the activity of the upstream promoter carrying a mutated p53 response element (p53RE) was determined.

Since TFIIA-binding ability of TLP was found to affect the transcriptional activation function of the upstream promoter (Fig. 2C,D), we investigated how TFIIA works for *p21* promoters. Overexpression of TFIIAαβ considerably stimulated the upstream promoter (Fig. 3A-a, lane 3). Since TFIIAγ exhibited little effect (Fig. 3A-a, lane 4), the concentration of TFIIAγ in cells seemed to be sufficient for the upstream promoter. We further investigated the cooperative effect directed by TLP and TFIIA in transcriptional regulation through co-overexpression of TFIIAαβ and TLP or N37E. Although the activation degree of TLP for the upstream promoter was 2.6-fold, co-overexpression of both TLP and TFIIAαβ yielded 4.0-fold activation (Fig. 3A-b, lanes 1–3). However, this additive effect was relatively small (1.8-fold) when N37E and TFIIAαβ were used (Fig. 3A-b, lanes 4 and 5). A dose-responsive effect of TFIIA on TLP-dependent promoter activation was observed (Fig. 3A-c), suggesting physical and functional interactions between TLP and TFIIA. To exclude a possibility that overexpressed TFIIA increases the amount of TLP protein, we examined the expression level of TLP and TFIIA and confirmed that TFIIA does not exhibit a significant effect on the expression of both endogenous and exogenous TLP (Fig. 3A-d). The downstream promoter was potentiated only slightly by TFIIA (Fig. 3B). Therefore it is suggested that the upstream promoter is much more sensitive to the concentration of TFIIA and that TFIIA-binding activity of TLP is involved in this process. Knockdown experiments revealed that the upstream promoter is much more dependent on TFIIA than is the downstream promoter (Fig. 3C–a). The effect of downregulation of TFIIA by short interfering RNA (siRNA) was evaluated by western blotting (Fig. 3C–b). We then investigated whether TFIIA-dependent activation of the upstream promoter occurs in p53-deficient cells. As shown in Fig. 4A, however, TFIIA did not activate the upstream promoter in p53-deficient cells. Moreover, the upstream promoter harboring a mutant p53RE did not respond to TFIIA in addition to TLP (Fig. 4B).

### Recruitment of TFIIA to the upstream promoter of the endogenous *p21* gene

**Fig. 5:**
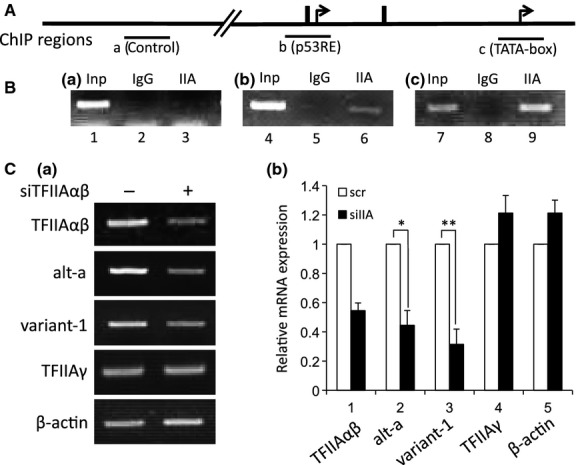
Functional association of TFIIA with the upstream promoter. (A) Schematic illustration around the *p21* upstream region. Three regions indicated by horizontal lines (a, control region; b, p53RE-containing upstream promoter; c, TATA-box-containing downstream promoter) were assayed by ChIP. (B) Determination of chromatin-bound TFIIA by ChIP assay with TFIIAαβ-specific antibody. (C) Effect of TFIIA knockdown on *p21* gene expression. TFIIAαβ was depressed by specific siRNA, and alt-a, variant-1 and several control RNAs were determined by RT-PCR (a) and RT-qPCR (b). Each mRNA level for scr (open column) is assigned as 1.0, and relative mRNA levels for siIIA (solid columns) are shown.

It has been reported that TFIIA can be recruited to some TATA-less promoters as well as TFIID-dependent TATA-containing promoters [26,27,30,31]. In this study, we demonstrated that TFIIAαβ activates the upstream promoter additively with native TLP (Fig. 3A-b,c). We investigated whether TFIIA is associated with *p21* promoters. Although we detected chromatin-bound TFIIA in the upstream promoter region (p53RE) as well as the downstream promoter region (TATA-box) (Fig. 5B), the amount of TFIIA was larger for the downstream promoter (Fig. 5B-c), possibly due to TFIID-assisted recruitment. We performed TFIIA knockdown experiments to examine TFIIA function for *p21* gene regulation, and we found that the production of alt-a mRNA was dependent on the amount of TFIIA (Fig. 5C). The amount of p21 variant-1 (Fig. 5C) and the total amount of *p21* mRNA (data not shown) were also decreased in TFIIA-depressed cells.

### Recruitment of TFIIA and TFIIA-reactive TLP to the upstream promoter in etoposide-treated cells

**Fig. 6:**
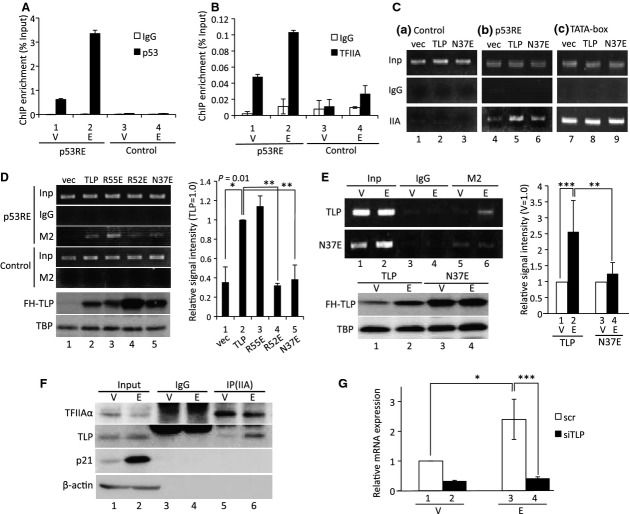
Recruitment of TFIIA and TLP to the *p21* upstream promoter upon etoposide stress. Etoposide-induced recruitment of p53 (A) and TFIIA (B) to the upstream promoter. Normal HCT116 cells treated with etoposide were subjected to ChIP assay using specific antibodies. ChIP enrichment was determined by qPCR. (C) Amount of chromatin-bound endogenous TFIIA was determined by ChIP using α-TFIIAαβ antibody. Cells transfected with an effector plasmid (TLP or N37E) and empty vector (vec) were treated with etoposide, and chromatin-bound TFIIA was detected for three regions as shown in Fig. 5A. (D) Binding of mutant TLPs to the upstream promoter of the endogenous *p21* gene. Cells into which FH-tagged wild-type and mutant TLP had been introduced were subjected to ChIP assay using α-FLAG M2 beads after etoposide treatment. Results are shown in the left panel. p53RE, an experimental p53RE-containing upstream promoter region; control, negative control region. Nuclear TBP and exogenous TLP proteins were determined by western blotting. We assigned the signal intensity of TLP (lane 2) as 1.0, and relative intensities of experimental ChIP signals are displayed as ratios to that of each TLP (right panel). (E) Amount of chromatin-bound TLP in etoposide-treated cells. Enrichment of chromatin-bound TLP or N37E at the upstream promoter region was determined. Cells treated with etoposide (E) or a solvent (V) were subjected to ChIP (left panel). Nuclear TBP and exogenous TLP proteins were determined by western blotting. Relative intensity of experimental ChIP signals (E/V for each TLP protein) is also shown (right panel). (F) Association of TFIIA and TLP in etoposide-treated cells. Cells treated with etoposide or a solvent were harvested and a co-immunoprecipitation assay was performed. Material immunoprecipitated with α-TFIIAαβ antibody was detected for TLP. IP(IIA), immunoprecipitation with α-TFIIAαβ. (G) Requirement of TLP for etoposide-stimulated expression of the *p21* gene. HeLa cells transfected with TLP siRNA or control siRNA were exposed to etoposide. The level of alt-a was determined by RT-qPCR. We assigned the mRNA level of lane 1 as 1.0, and the relative mRNA level of each sample is shown.

Previously, we demonstrated that p53 and TLP are recruited to the same region of the *p21* gene in etoposide-treated cells [14]. In this study, we confirmed that p53 (Fig. 6A) and TLP (Fig. 6D, lanes 1 and 2) were substantially recruited to the upstream promoters in cells treated with etoposide, which is one of the typical genotoxins. Figure 6B shows that large amounts of TFIIA bind to the upstream p53RE-containing region. Furthermore, we found that exogenous TLP but not N37E increased the amount of upstream promoter-bound TFIIA (Fig. 6C). These results indicate that TFIIA-binding ability of TLP is required for recruitment of TFIIA to the upstream promoter. We next determined how many mutant TLPs are recruited to the promoter in etoposide-treated cells. Exogenously expressed TLP and R55E clearly bound to the p53-responsive element (Fig. 6D, lanes 2 and 3). On the other hand, R52E and N37E showed decreased binding signals (lanes 4 and 5) although these proteins were substantially present in cells. A further chromatin immunoprecipitation (ChIP) assay demonstrated that the amount of promoter-bound wild-type TLP was significantly increased by etoposide, whereas that of N37E did not change and was lower than TLP, even though amounts of N37E were higher than those of TLP in nuclei of control and etoposide-treated cells (Fig. 6E). Consequently, R52 and N37E, whose chromatin-binding results were overestimated (Fig. 6D,E), were demonstrated to have weakened promoter-binding abilities compared with wild-type TLP. We then examined the effect of etoposide on TLP–TFIIA interaction. A co-immunoprecipitation experiment revealed that TLP and TFIIA form an intracellular complex in etoposide-treated cells upon DNA damage (Fig. 6F). The same result was obtained when exogenously expressed FH-TFIIA was examined (data not shown).

Lastly, we investigated whether activity of the upstream promoter is modulated by TLP in etoposide-treated cells. TLP knockdown resulted in a decrease in upstream promoter-driven mRNA production (Fig. 6G, left two columns). We found that the net quantity of knockdown-directed decreased mRNA production in etoposide-treated cells was twice as much as that obtained in normal cells (Fig. 6G, right two columns). We have confirmed that etoposide enhances transcription from the endogenous *p21* upstream promoter [14,23]. These situations suggest that etoposide-augmented promoter-recruited TLP, perhaps together with TFIIA, works for activated transcription from the *p21* upstream promoter.

### Negative regulation of cell growth by TLP and TFIIA

**Fig. 7:**
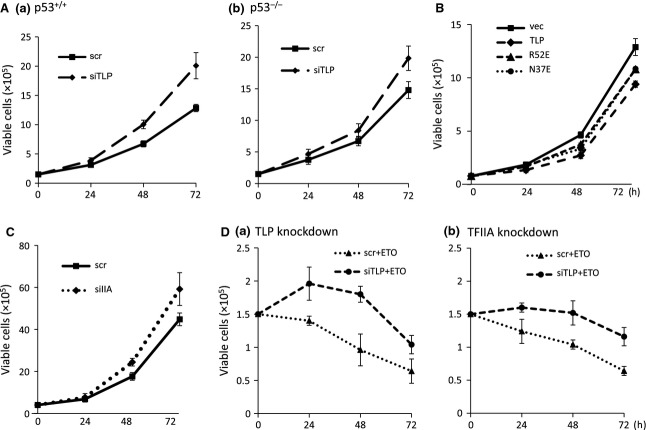
Inhibitory effect of TLP and TFIIA on cell growth. HCT116 cells were treated with dimethylsulfoxide (A) or etoposide (D). (A) Normal (a) and *p53*^−/−^ cells (b) were transfected with TLP siRNA (siTLP) or control siRNA (scr). The cells were replated and cultured. Then cell numbers were counted at the indicated times. (B) Growth profile of TLP-overexpressing cells. Cells transfected with an effector plasmid expressing TLP or its mutants were replated and cell numbers were counted. (C) Growth profile of TFIIAαβ knockdown cells. Cells were transfected with TFIIAαβ siRNA (siIIA) or control siRNA (scr), and the growth profile was analyzed. (D) Knockdown of TLP (a) or TFIIA (b) of etoposide-treated cells. Cells transfected with siRNAs were cultured in an etoposide-containing medium and viable cells were counted at the indicated times.

In the above-described figures, we showed that TLP and TFIIA potentiate the upstream promoter of the *p21* gene and upregulate anti-mitotic p21 protein. We then examined how TLP and TFIIA affect the growth profile. Knockdown experiments demonstrated that reduction of endogenous TLP resulted in elevated proliferation for both normal (Fig. 7A-a) and p53-deficient cells (Fig. 7A-b). However, the rate of proliferation acceleration caused by depression of TLP was much higher for wild-type cells than in p53-deficient cells. Moreover, overexpression of N37E exhibited a slight but significant growth-inhibitory effect compared with wild-type TLP (Fig. 7B). Knockdown of TFIIA also resulted in acceleration of the cell proliferation rate (Fig. 7C). We next examined the effects of TLP and TFIIA on the profile of etoposide-triggered cell death by knockdown experiments (Fig. 7D), and we found that both TLP (Fig. 7D–a) and TFIIA (Fig. 7D–b) accelerated cell death rate in a DNA-damaged condition. Since TLP and TFIIA play a negative role in cell growth and since association of the two factors is implicated from the results shown in Fig. 7B, these factors might modify the expression of growth- and apoptosis-related genes including *p21*.

## Discussion

Previously, we demonstrated that TLP, which is one of the TBP family proteins, is involved in regulation of the upstream promoter [14]. The most attractive property of TLP is its stronger TFIIA-binding ability than that of TBP [29,30], although the functional significance of this property has not been elucidated. Bryant *et al*. [31] reported that mutant TBPs with decreased TFIIA-binding ability showed decreased transcription activation function *in vitro*. In this study, we demonstrated that Asn37 and Arg52 of TLP, which correspond to TFIIA-reactive Asn189 and Arg205 of TBP, respectively [31], are required for TFIIA binding in human cells (Fig. 1). N37E and R52E exhibited decreased transcriptional activation functions for the endogenous upstream promoter, while R55E, which has substantial TFIIA-binding capacity, exhibited native function (Fig. 2C). These mutant TLPs also exhibited decreased transcriptional activation function for the upstream promoter in an exogenous reporter plasmid (Fig. 2D). Consequently, TFIIA-binding ability of TLP is thought to be required for TLP-dependent transcriptional activation. Although R52E had binding ability to processed TFIIA (Fig. 1, lane 12), it exhibited little transcriptional function. Because unprocessed TFIIA has been reported to be transcriptionally active [28], it is possible that binding to unprocessed TFIIA is required for TLP function to activate *p21* upstream promoter.

TFIIA activates RNA polymerase II promoters via interaction with various transcription factors. As is generally known, TFIIA indirectly associates with the TATA-box promoter element via TBP [1–3,6]. Li *et al*. [24] showed that TFIID is recruited to the TATA-box of the *p21* downstream promoter and that p53 is associated indirectly with the TATA-box via TFIID. Indeed, abundant chromatin-bound TFIIA was detected at the downstream promoter (Fig. 5B). However, overexpressed TFIIA enhanced the endogenous downstream promoter only slightly (Fig. 3B). On the other hand, the upstream promoter was significantly activated by TFIIA (∼3.0-fold) (Fig. 3A-a). Moreover, results shown in Fig. 3C and maybe Fig. 5C suggest that the upstream promoter requires a high concentration of intracellular TFIIA for its maximal activity. It has remained a question for a long time why TFIIA is an essential factor for cell growth [32], despite the fact that it works just as a cofactor. We speculate that some essential TATA-less genes need TFIIA as well as TLP. Results shown in Fig. 3A-b also demonstrate an additive effect between TFIIA and TFIIA-interactive TLP but not mutant ones, suggesting a functional interaction of these two factors for the upstream promoter. As already stated, the human *p21* gene has two major promoters: a TATA-less upstream promoter and a TATA-containing downstream promoter [23,24]. Although the mechanism by which TFIIA exhibits different responses to the two promoters of the *p21* gene is not fully understood, the TATA-box element seems to be one of the determinants. Existence of multiple promoters of the *p21* gene might have an advantage to maximize the level of gene expression, which is governed by different sets of transcription factors, when cells are exposed to different kinds of stimuli and stresses.

It has been confirmed that the upstream promoter is basically driven by p53 [23]. Activity of the upstream promoter was almost inert in p53-deficient cells (Fig. 4A). Moreover, TLP does not exhibit a transcription activation function for the upstream promoter in p53-null cells. We found that the upstream promoter is upregulated by TFIIA and is dependent on TFIIA in addition to p53 and TLP (Figs 3 and 4). The results shown in Figs 5 and 6 demonstrate that these three transcription factors are recruited to the upstream promoter, and etoposide, which stimulates *p21* gene expression, increased this recruitment. *Drosophila* TLP works as a cofactor for DREF transcription factor of the *PCNA* gene [13]. Moreover, TFIIA can work as a co-activator of several activators [33–36] and binds to p53 [37]. We have observed intracellular binding of TLP and TFIIA [29]. Furthermore, we showed interaction between TLP and p53 [25]. We therefore speculate that TLP can form a triple complex with TFIIA and p53, and TLP and TFIIA coordinately function as a binary co-activator complex for p53 on the *p21* upstream promoter. The fact that the native, but not N37E, TLP stimulates the upstream promoter additively with TFIIA (Fig. 3A-b) supports this hypothesis.

In addition to p21, TLP and p53 are widely involved in growth repression and apoptosis of cells. The present study revealed that TFIIA is also associated with the function of TLP. The results presented in Fig. 7A show that TLP-mediated growth repression is dependent on p53. Since TLP with decreased TFIIA reactivity exhibited a weaker growth-inhibitory effect (Fig. 7B), some parts of TLP-mediated growth repression can be governed by at least TLP- and TFIIA-dependent transcription from the upstream promoter of the anti-mitotic *p21* gene. Furthermore, we observed that TLP and TFIIA are also involved in etoposide-mediated cell death (Fig. 7D). We believe that TLP and TFIIA contribute to this phenomenon through interaction with the *p21* upstream promoter.

## Materials and methods

### Cell culture, drug treatment and DNA transfection

Human HCT116 cells (wild-type and p53-deficient mutant cells) [27] and HeLa cells were maintained in Dulbecco's modified MEM with high glucose and low glucose respectively (Sigma-Aldrich, St. Louis, MO USA) at 37 °C in the presence of 10% fetal bovine serum. Cell numbers were counted by the trypan blue dye-exclusion method with a hematocytometer. Etoposide dissolved in dimethylsulfoxide was added to the medium to 30–50 μm. Transfection of nucleic acids was performed by using Lipofectamine and Plus Reagent (Invitrogen, Carlsbad CA, USA).

### Expression plasmids for mammalian cells

pCIneo-FH-TLP, which is an expression plasmid of flag/oligohistidine (FH) tagged mouse TLP, was described previously [14]. Mouse and human TLPs have an identical amino acid sequence. Plasmids for mutant TLPs (R55E, R52E and N37E) were described previously [29]. TFIIA expression plasmids, pCIneo-FH-TFIIAαβ and pCIneo-FH-TFIIAγ, have an open reading frame of human TFIIAαβ and TFIIAγ with an FH-tag at their amino termini.

### Reporter plasmids for luciferase assay

pGL4.10 vector (Promega, Madison WI, USA) was used for construction of luciferase reporter plasmids. A reporter plasmid (designated p21up/GL4) containing a human *p21* promoter region encompassing from −2266 to −1875 was described previously [14]. The +1 position represents the transcription start site of the downstream promoter. In this study, we constructed two new luciferase reporter plasmids, p21down/GL4 and p21core/GL4, that contain a downstream promoter region from −168 to +66 and a short DNA stretch from −5 to +66 of the *p21* downstream promoter, respectively. These constructs were generated by a PCR-based strategy using a reporter plasmid encompassing from −2677 to +66, which has been named p21luc1 as previously described [14]. Primer sets to amplify DNA fragments from −168 to +66 and from −5 to +66 sequences were as follows: −168 to +66 forward, 5′-CTCGAGGGCCTGCTGGAACTCGGCCAG; −5 to +66 forward, 5′-CTCGAGGCGCCAGCTGAGGTGTGAGCA; and common reverse, 5′-AGATCTCGGCGAATCCGCGCCCAGCT.

### RNA interference

siRNAs were prepared by a Silencer siRNA Construction Kit (Ambion, Carlsbad, CA, USA). Sequences for target human TFIIAαβ were 5′-GATGGGCAGGTGGAAGAAG (sense) and 5′-CTTCTTCCACCTGCCCATC (antisense). The sequence for human TLP was described previously [14]. A scrambled sequence of a part of TFIIAαβ was used as a control siRNA. Cells were transfected with 50–100 nm of siRNA and cultured for an appropriate period.

### PCR

Total cellular RNAs were prepared using an RNeasy Mini Kit (Qiagen, Chatsworth, CA, USA), and RT-PCR was performed as described previously [38]. Amplified products were analyzed by agarose gel electrophoresis. Quantitative determination of the PCR products (qPCR) was performed using a Thunderbird qPCR Mix (Toyobo, Osaka, Japan) and 7300 Real Time PCR System (Applied Biosystems, Foster City, CA, USA). All reactions were performed in triplicate. Primer sets to detect p21 transcripts were as follows: total p21 forward, 5′-GACACCACTGGAGGGTGACT; reverse, 5′-CCCTAGGCTGTGCTCACTTC; alt-a forward, 5′-GGTGGCTATTTTGTCCTTGG; reverse, 5′-ACAGGTCCACATGGTCTTCC; variant-1 forward, 5′-CTGCCGAAGTCAGTTCCTTG; reverse, common to alt-a reverse.

### Luciferase assay

Cells were inoculated into a 24-well plate (8 × 10^4^cells·well^−1^). Twenty-four hours later, cells were transfected with the indicated amount of a reporter plasmid and an effector plasmid and cultured for 24 h. Total amounts of transfected DNA were adjusted with pRL-TK (Promega). Cells were disrupted with a Passive Lysis Buffer (Promega). Luciferase activity in lysates was determined by a Dual Luciferase Reporter Assay System (Promega).

### Immunoprecipitation of intracellular proteins

Cell extracts were prepared as previously described [29]. Five hundred micrograms of the extract was used for immunoprecipitation. Endogenous proteins in extracts were mixed with a specific antibody and precipitated with protein G-Sepharose 4 Fast Flow (GE Healthcare Bioscience, Piscataway, NJ, USA). FH-proteins in extracts were precipitated by anti-Flag M2 Affinity Gel (Sigma-Aldrich). Normal rabbit IgG (Santa Cruz, Santa Cruz, CA, USA) and IgG-Sepharose 6 Fast Flow (GE Healthcare Bioscience) were used as control antibodies. Bound proteins were eluted and analyzed by western blotting as described before [29].

### Western blotting

Proteins were separated by SDS/PAGE, transferred to an Immobilon-P poly(vinylidene difluoride) membrane (Millipore, Billerica, MA, USA) and detected by an ECL Prime (GE Healthcare Bioscience) as previously described [29] by using specific antibodies and appropriate horseradish-peroxidase-conjugated secondary antibodies including anti(α)-rabbit IgG and α-mouse IgG. As primary antibodies, we used α-p53 antibody (Santa Cruz), α-β-actin antibody (Sigma-Aldrich) and antigen-purified α-TLP antibody and TFIIAαβ antibody as described previously [29].

### Chromatin immunoprecipitation (ChIP)

Cells transfected with plasmids were treated with 50 μm etoposide for an appropriate time. After fixation of cells, ChIP assay was performed as described previously [14]. Endogenous and exogenous FH-proteins were precipitated with a specific antibody and Protein G-Sepharose 4 Fast Flow (GE Healthcare Bioscience) and α-Flag M2 Affinity Gel (Sigma-Aldrich), respectively. Immunoprecipitated DNAs and control input DNAs were analyzed by semi-quantitative PCR or qPCR using *p21* promoter-specific primer sets. Primer sets for ChIP analysis were as follows: p53RE forward, 5′-CACCTTTCACCATTCCCCTA; reverse, 5′-GCAGCCCAAGGACAAAATAG; TATA-box forward, 5′-TGCTGGAACTCGGCCAGGCTCAGCTG; reverse, 5′-CCAGCTCCGGCTCCACAAGGAACTG; control forward, 5′-TGGTAGGCCTCTCCAAGGTA; reverse, 5′-ACACATGTGACTTGGGGTGA.

### Statistical analysis

Data in this study are shown as mean ± standard error of the mean obtained from at least three independent experiments. Statistical significance of quantitative data was determined using Bonferroni's method with r console (ver. 3.0.3). The number of experiments used for statistical analysis was at least three (*n* = 3). *P* < 0.05 was considered to be statistically significant. Statistical significance of differences between samples is shown in the figures with asterisks: *P < 0.05; **P < 0.01; ***P < 0.001.

## Abbreviations

ChIP: chromatin immunoprecipitation
qPCR: quantitative PCR
siRNA: short interfering RNA
TBP: TATA-binding protein
TFIIA: transcription factor IIA
TLP: TBP-like protein
